# Zika Virus Outbreak, Bahia, Brazil

**DOI:** 10.3201/eid2110.150847

**Published:** 2015-10

**Authors:** Gubio S. Campos, Antonio C. Bandeira, Silvia I. Sardi

**Affiliations:** Federal University of Bahia, Salvador, Bahia, Brazil (G.S. Campos, S.I. Sardi);; Hospital Aliança, Salvador (A.C. Bandeira)

**Keywords:** Zika virus, viruses, outbreak, maculopapular rash, detection, diagnosis, reverse transcription PCR, Bahia, Brazil

**To the Editor:** Zika virus (ZIKV) is a mosquito-borne flavivirus related to yellow fever virus, dengue virus (DENV), and West Nile virus (WNV). It is a single-stranded positive RNA virus (10,794-nt genome) that is closely related to the Spondweni virus and is transmitted by many *Aedes* spp. mosquitoes, including *Ae. africanus*, *Ae. luteocephalus*, *Ae. hensilli*, and *Ae. aegypti*. The virus was identified in rhesus monkeys during sylvatic yellow fever surveillance in the Zika Forest in Uganda in 1947 and was reported in humans in 1952 (*1*).

In 2007, an outbreak of ZIKV was reported in Yap Island, Federated States of Micronesia (*2*). ZIKV also caused a major epidemic in the French Polynesia in 2013–2014 (*3*), and New Caledonia reported imported cases from French Polynesia in 2013 and reported an outbreak in 2014 (*4*).

A new challenge has arisen in Brazil with the emergence of ZIKV and co-circulation with others arboviruses (i.e., DENV and chikungunya virus [CHIKV]). We report ZIKV infection in Brazil associated with a recent ongoing outbreak in Camaçari, Bahia, Brazil, of an illness characterized by maculopapular rash, fever, myalgias/arthralgia, and conjunctivitis.

On March 26, 2015, serum samples were obtained from 24 patients (Table) at Santa Helena Hospital in Camaçari who were given a presumptive diagnosis of an acute viral illness by emergency department physicians. These patients were given treatment for a dengue-like illness, and blood samples were obtained for complete blood counts and serologic testing by using an ELISA specific for IgG and IgM against DENV.

Serum samples were analyzed at the Federal University of Bahia by reverse transcription PCR (RT-PCR) to detect DENV, CHIKV, WNV, Mayaro virus, and ZIKV. In brief, serum samples were subjected to RNA extraction by using the QIAamp Viral RNA Mini Kit (QIAGEN, Hilden, Germany). RNA was reverse transcribed by using the SuperScript II Reverse Transcription Kit (Invitrogen, Carlsbad, CA, USA) and subjected to PCRs specific for DENV (*5*) CHIKV (*6*), WNV (*7*) and Mayaro virus (*8*). A positive RT-PCR for a partial region of the envelope gene with primers ZIKVENF and ZIKVENVR (positions 1538–1558 and 1902–1883, respectively) (*9*) was considered indicative of ZIKV infection. PCR products (362 bp) were sequenced at the ACTGene Analises Moleculares, Alvorada, Rio Grande do Sul (Porto Allegre, Brazil), and sequences were deposited in GenBank under accession nos. KR816333–KR816336.

All patients were negative by RT-PCR for DENV, Mayaro virus, and WNV. Samples from 7 (29.2%) patients were positive by RT-PCR for ZIKV (369-bp fragment) and from 3 (12.5%) patients for CHIKV (305-bp fragment). There was no simultaneous detection of ZIKV and CHIKV. Most (85.7%) patients positive for ZIKV were women; they had a median age of 28 years and no history of international travel. Patients positive for ZIKV sought medical care after a 4-day (range 1–5 days) history of rash, myalgias, arthralgias, or fever. Three patients had IgG against DENV, which is consistent with a previous DENV infection, and none of the 7 ZIKV-positive patients had a positive response for DENV.

Mean laboratory findings for patients with acute ZIKV infection were a leukocyte count of 3,750 cells/mm^3^ (range 2,790 cells/mm^3^–6,150 cells/mm^3^) and a platelet count of 180,000 platelets/mm^3 ^(range 151,000 platelets/mm^3^–274,000 platelets/mm^3^). The mean C-reactive protein level was 16.3 mg/L (range 0.9 mg/L–19.7 mg/L). Sign and symptom duration was 1–5 days, and most patients had a maculopapular rash, myalgias, fever, and headache. Arthralgia was seen less frequently.

ZIKV infections were assessed by sequencing partial ZIKV envelope gene regions of isolates. Phylogenetic analysis rooted with Spondwei virus showed that ZIKV sequences obtained belonged to the Asian lineage and showed 99% identity with a sequence from a ZIKV isolate from French Polynesia (KJ776791) (*10*).

We report ZIKV infection in Brazil in association with an ongoing outbreak of an acute maculoexantematic illness. Although the patient population samples were not randomly selected, 42% (10/24) of the patients were positive for ZIKV (n = 7) or CHIKV (n = 3) and had maculopapular rash, fever, myalgias and headache. After detection of ZIKV in Bahia, many cases have been identified in other states (http://www.promedmail.org, archive no. 20152015602.343.1158).

Cases of infection with DENV, CHIKV, and ZIKV in Brazil and elsewhere will make diagnosis based on clinical and epidemiologic grounds unreliable. These issues show the need for laboratory confirmation of these arboviral infections. More studies are needed to address the effects of these concurrent arboviruses infections in Brazil.

**Table:** Characteristics of 24 patients with positive and negative results for infection with Zika virus, Brazil, 2015

Reverse transcription PCR result for Zika virus (no.)	Mean (SD) patient age, y	Patient sex, F/M	No. (%)			
			Rash	Fever	Myalgia	Headache
Positive (7)	33 (15)	6/1	6 (85.7)	3 (43)	4 (57.1)	3 (43)
Negative (17)	31 (8.5)	12/5	12 (70.6)	6 (35.3)	9 (53)	11 (64.7)
